# Dentists’ attitudes toward patient-centered care and its predictors: a cross-sectional study in South Korea

**DOI:** 10.1186/s12903-023-02791-9

**Published:** 2023-02-06

**Authors:** Minjung Lee, Youngha Song, Myoungsoon You, Shin-Young Park, Jungjoon Ihm

**Affiliations:** 1 Dental Research Institute, School of Dentistry, Seoul National University, Seoul, Korea; 2 Department of Preventive and Social Dentistry, Seoul National University, Seoul, South Korea; 3 Department of Public Health Sciences, Seoul National University, Seoul, South Korea; 4 Department of Dental Education, Seoul National University, Seoul, South Korea; 5 Predoctoral Treatment Center, Seoul National University Dental Hospital, Seoul, South Korea; 6 Interdisciplinary Program in Cognitive Science, Seoul National University, Seoul, Korea

**Keywords:** Patient-centered care, Patient-centered attitudes, Empathy, Dentists, South Korea

## Abstract

**Background:**

Patient-centered care (PCC) has been one of medical practice’s most frequently discussed principles. However, attitudes toward PCC among dentists remain underexplored. This study focuses on examining dentists’ patient-centered attitudes and investigating their predictors.

**Methods:**

The Patient–Practitioner Orientation Scale which consists of Sharing and Caring subscales was used to assess patient-centered attitudes. The statistical analysis included 217 dentists from South Korea. Hierarchical linear regression analysis was performed to examine the predictors such as sociodemographic aspects, academic factors, work-related factors, and empathy.

**Results:**

A patient-centered attitude of Caring subscale (M = 4.29, SD = 0.56) emerged, but the provider-centered attitude was higher in Sharing subscale (M = 3.40, SD = 0.48). Work year, academic track, and empathy were associated significantly with an overall caring aspect of patient-centered attitude, while the gender effect remained insignificant. Empathy had a critical and significant impact on the patient-centered attitude.

**Conclusions:**

Efforts to enhance patient-centeredness in Sharing are needed; post-graduate education and transition to a more patient-centered health system are recommended. Moreover, empathy still matters as it was found to be a significant predictor of patient-centered attitudes. The findings of this study support the need for efforts to enhance patient-centered attitudes among dentists, which will help generate discussion on improving the curriculum of post-graduate education and health system reform.

**Supplementary Information:**

The online version contains supplementary material available at 10.1186/s12903-023-02791-9.

## Background

In recent decades, patient-centered care (PCC) has become one of medical practice’s most frequently discussed principles. Various conceptual models of PCC commonly describe patient-centeredness as the opposite of disease-centered or provider-centered care. In contrast to ‘disease-centered,’ “patient-centered” emphasizes the content of the consultation and the choice of topics to be addressed, reflecting patients’ needs and expectations [1]. When considering “patient-centered” as opposed to “provider-centered,” it deals with the balanced control/power and the consideration of patients’ perspectives in healthcare encounters [2]. Patient participation in decision-making to reach a consensus that the patient and the doctor can agree upon is emphasized [3].

PCC is crucial for dental patient experience and oral health outcomes [4–6]. Dental patients often experience negative emotions such as anxiety and fear [7] and PCC was associated with lower fear and higher patient satisfaction [8–10]. Interviews with patients with dental phobia revealed that dentist understanding and accepting patient needs and concerns were more important than technical competence [11]. Moreover, PCC can encourage dental patients to participate in decision-making and check their willingness and ability to follow treatment plans [12, 13], and help to ensure that patients are held accountable and compliance is enhanced [14, 15].

There is an essential distinction between dental and medical encounters. First, dental consultation includes interviews for investigation, presenting a diagnosis, prescribing treatments, and giving advice to the patient. However, actual dental treatment is also performed at the same time. This makes a significant difference from medical consultation; while dentists aim to provide efficient care, patients often expect unpleasant events on the spot. Second, by its verbal nature, dentistry physically limits a patient’s ability to communicate verbally during the therapeutic phase of consultation. Therefore, different approaches from medical consultation are required for dental consultation [16]. Dentistry can benefit from the findings in PCC from the medical context, however, applying it to the dental setting can be misleading given the differences in settings [6, 13, 16]. PCC in dentistry remains unexplored and has only recently emerged [17].

The present study addresses whether dentists favor PCC and which factors affect this patient-centered attitude. We tested the association between patient-centered attitude and factors such as gender [18–23] and years of medical education [18, 20, 24] which have shown to be related to more patient-centered attitudes in previous studies. Empathy, “the ability to understand the patient’s situation, perspective, and feelings, and to communicate that understanding to the patient” [25], also influences attitudes toward PCC, as an essential element of PCC and patient-centered communication [20]. A positive association emerged between empathy and patient-centered attitudes among medical [26, 27] and dental students [28, 29]. Specifically, this study (1) investigates attitudes toward PCC of dentists in Korea, (2) explores dentists’ characteristics that are possibly associated with patient-centered attitudes, and (3) examines the influence of empathy on patient-centered attitudes. Implications for developing interventions such as patient-centered education programs and policies to enhance PCC in dentistry are also discussed in this paper.

## Methods

### Study design

A cross-sectional online survey was developed to (a) evaluate the dentists’ patient-centered attitude and empathy and (b) assess the association between attitude toward PCC and empathy. The survey was conducted via an online platform with Google survey. The Korean Government has asked the public to minimize face-to-face interaction potential respondents were electronically invited to participate (October 11, 2021). An invitation to participate in the study including a brief introduction on the background, the objective of study, voluntary nature of participation, declarations of confidentiality and anonymity was sent via e-mail to dentists (n = 500) who practice in metropolitan area and had registered with the Korean Dental Association (KDA) [40]. Participation was voluntary, and informed consent was obtained from all respondents before taking the survey. The sample size was calculated using G*Power (latest ver. 3.1.9.7; Heinrich-Heine-Universität Düsseldorf, Düsseldorf, Germany) to calculate the sample size. The required calculated sample size was 216 with a confidence level of 95% and a 5% margin of error. The response acceptance was closed (November 12, 2021) when the required sample size was achieved. This study was approved by the Institutional Review Board of the School of Dentistry, the Seoul National University, Seoul, Republic of Korea, as per the policy on research with human participants (Institutional Review Board No. S-D20200028).

### Study instruments

#### Patient–Practitioner Orientation Scale (PPOS)

The Patient-Practitioner Orientation Scale (PPOS) is a measure often used to quantify the roles that healthcare professionals and patients believe they should play in their interactions regarding patient-centeredness [30, 31]. Research has provided evidence to support the construct validity of the PPOS scores, and the PPOS has been used efficaciously in a variety of medical education and practice contexts also non-Western cultures [18, 28, 32, 33]. The measure uses two subscales, “caring” (i.e., understanding the patient’s perspective) and “sharing” (i.e., sharing responsibility and authority in decision-making with the patient). Some samples include the following: “Patients should be treated as if they are partners of the doctor, equal in power, and status” (Sharing subscale), “When doctors ask a lot of questions about a patient’s background, they are prying too much into personal matters” (Caring subscale). The PPOS questionnaire was translated into Korean and validated [34, 35]. The responses were rated on a 6‐point Likert-type scale of 1 = strongly agree to 6 = strongly disagree. All items were written in a provider-centered style, but three items were written in a patient‐oriented style; therefore, scoring was reversed, so a higher score indicates a patient‐oriented style.

#### Interpersonal reactivity index (IRI)

The interpersonal reactivity index (IRI) measures individual differences in empathy in the general population and contains 28 items with response options on a 5-point Likert scale (0 = does not describe me very well to 4 = describes me very well) [36, 37]. IRI has a multidimensional approach to empathy, which is known to be associated with patient-centered attitude. Empathy is measured by ‘cognitive subscales’: (1) Perspective taking (IRI-PT), which measures the tendency to spontaneously adopt the psychological point of view of others, and (2) Fantasy (IRI-FS), which measures the ability to transform oneself into characters of movies, books, plays, etc., and ‘affective aspects’, which can be measured using two affective subscales: (3) Empathic concern (IRI-EC) that measures the ability to assess ‘other'-oriented feelings of sympathy and concern for unhappy others and (4) Personal distress (IRI-PD) that measures “self” oriented feelings of distress and unease in interpersonal settings [36]. This study used the Korean version of the IRI (K-IRI) translated and validated by Kang et al. [39]. This K-IRI has been used to assess empathy in Korean dental students with the results of appropriate psychometric properties [28]. For a full list of measures, see Additional File 1.

### Statistical analysis

All statistical analyses were performed using R version 3.5.1 (R Foundation for Statistical Computing, Vienna, Austria). All the results of quantitative variables were reported either as mean (M), standard deviation (SD), or frequency (percentage %). We conducted an item-by-item analysis of PPOS by calculating the proportion of the sample that agreed with each statement (combining the proportion that responded, ‘strongly agree’ or ‘mostly agree,’ and ‘agree’). Items were analyzed in the direction they were initially presented to participants, with higher scores consistently representing stronger disagreement with the statement. Composite scores for the full scale and subscale of PPOS have a possible range of 1–6, with higher values indicating higher patient-centeredness. To determine individual background factors’ role in patient-centered attitudes, differences in socio-demographics, academic characteristics, and work-related factors were compared with the patient-centered attitude using the t-test and Analysis of variance (ANOVA) test statistics. Multivariate hierarchical linear regression analysis was used to examine the effect of sociodemographic aspects, academic factors, work-related factors, and empathy on patient-centered attitude.

## Results

### Descriptive analysis

We collected surveys from 223 dentists who gave informed consent to participate in the survey (response rate 44.6%), and 217 were included in the analysis after excluding responses with missing values. A sample of 217 dentists included 137 males (63.1%) and 80 females (36.9%), with a mean age of 37 years (M = 37.26, SD = 7.34) (Table 1) [48]. About half of the respondents were in their 30 s (55.76%), followed by respondents in their 40 s (22.58%), 20 s (11.98%), and those over the age of 50 (9.68%). In Korea, dentistry school has been operating as a dental college (2 or 3 years pre-dental course and 4 years Doctor of Dental Surgery [DDS] degree program) and a professional graduate-entry school (4 years DDS degree program) system simultaneously. Students entering a professional graduate-entry school must have a bachelor’s degree in advance, but most students entering a dental college are high school graduates. A student from both tracks blends studies together to achieve the qualification of a dentist with a DDS degree. Among the study participants, 67.28% were dental college graduates, and 32.72% were professional graduate-entry school graduates. The average years of practice were 9.78 (SD = 7.20). Regarding practice ownership, 46.5% of the respondents were owners (n = 101), and 53.5% were associate dentists (n = 116). The dentists’ characteristics appear in Table 1.

**Table 1:** Descriptive statistics of survey respondents (n = 217)

Characteristics	No	%
*Gender*	217	
Male	137	63.13
Female	80	36.87
*Age groups*	M = 37.26	SD = 7.34
20–29	26	11.98
30–39	121	55.76
40–49	49	22.58
50 and older	21	9.68
*Academic track*		
Dental college	146	67.28
Professional graduate-entry school	71	32.72
*Years of practice (y)*	*M* = *9.78*	*SD* = *7.20*
< 5	56	25.81
5–10	74	34.10
10–15	35	16.13
15– 20	25	11.52
20<	27	12.44
*Workplace*		
Primary care clinic	179	82.5
Secondary/Tertiary care hospital	38	17.5
*Practice ownership*		
Owner/partner	101	46.5
Associate	116	53.5

Descriptive analyses were performed on patient-centered attitudes and empathy; these results appear in Table 2. The mean PPOS score across the sample was 3.85 (SD = 0.42), close to the midpoint of the possible range (1–6). Mean scores were slightly lower on the sharing subscale of 3.40 (SD = 0.48) and higher on the caring subscale of 4.29 (SD = 0.56) (Table 2). Cronbach’s alpha was high at the full scale (α = 0.74) and acceptable levels for the subscales of caring (α = 0.65) and sharing (α = 0.63). Higher PPOS values indicate a more patient-centered attitude regarding sharing power with patients and providing holistic patient care. Regarding empathy, among the four subscales, the empathic concern score was the highest (M = 3.54, SD = 0.61), followed by perspective taking (M = 3.50, SD = 0.57), fantasy (M = 3.27, SD = 0.65), and personal distress (M = 2.94, SD = 0.59). Cronbach’s alpha was high for the full scale (α = 0.83), the empathic concern subscale (α = 0.76), perspective taking (α = 0.72), fantasy (α = 0.78), and the personal distress (α = 0.72).

**Table 2:** Descriptive statistics for Patient–Practitioner Orientation Scale and empathy

Variable	M^a^	SD^b^
*Patient–Practitioner Orientation Scale (PPOS*^*a*^*)*		
PPOS (Overall)	3.85	.42
PPOS (Sharing)	3.40	.48
PPOS (Caring)	4.29	.56
*Empathy (IRI*^*b*^*)*		
IRI (Fantasy)	3.27	.65
IRI (empathic concern)	3.54	.61
IRI (perspective taking)	3.50	.57
IRI (personal distress)	2.94	.59

M^a^ Mean, SD^b^ Standard deviation

### Patient-centered attitude

Individual patient-centered attitude items were analyzed. Most dentists have shown a more provider-dominant style in seven of the nine sharing subscale items, as presented in Table 3. 83.9% of the respondents agreed, “The patient must always be aware that the dentist is in charge (item 15)”, and 82% agreed, “Patients generally want reassurance rather than information about their health (item 10).” However, 79.26% disagreed with the statement, “Patients should rely on their dentists’ knowledge and not try to find out their conditions on their own,” and 73.73% disagreed with, “Patients should be treated as if they were partners with the dentist, equal in power and status.” Conversely, only one of the nine caring subscale items was disease-centered: “The most important part of the standard dental visit is the physical exam” (70.0%). Most dentists have shown a more patient-centered style in caring for subscale items. For instance, 88.94% of the respondents disagreed with the statement “it is not that important to know a patient’s culture and background to treat the person’s illness,” and 88.48% disagreed with “if dentists are truly good at diagnosis and treatment, the way they relate to patients is not that important.” Differences in socio-demographics, academic factors, and work-related factors were compared with the patient-centered attitude using the* t*-test and Analysis of variance (ANOVA) test statistics in Table 4. A trend of increasing patient-centered attitude along age groups and years of practice has been found; females showed more patient-centered attitude; however, significant differences were not found.

**Table 3:** Descriptive statistics for Patient–Practitioner Orientation Scale and percent of dentists in disagreement with items

	M^a^	SD^b^	Disagree (%)	Orientation of sample
*Sharing*				
1. The doctor is the one who should decide what gets talked about during a visit	3.62	1.15	49.31	Provider-dominant
4. It is often best for patients if they do not have a full explanation of their medical condition	3.51	1.33	45.62	Provider-dominant
5. Patients should rely on their doctors’ knowledge and not try to find out their conditions on their own	4.41	1.12	79.26	Patient-centered
8. Many patients continue asking questions despite not learning anything	3.05	1.12	31.80	Provider-dominant
9. Patients should be treated as if they were partners with the doctor, equal in power and status	4.20	1.19	73.73	Patient-centered
10. Patients generally want reassurance rather than information about their health	2.53	0.99	17.97	Provider-dominant
12. When patients disagree with their doctor, this is a sign that the doctor does not have the patient’s respect and trust	3.58	1.37	48.85	Provider-dominant
15. The patient must always be aware that the doctor is in charge	2.57	1.13	16.13	Provider-dominant
18. When patients find out medical information on their own, this usually confuses more than it helps	3.17	1.07	32.72	Provider-dominant
*Caring*				
2. Although health care is less personal these days, this is a small price to pay for medical advances	4.18	1.19	70.97	Patient-centered
3. The most essential part of the standard dental visit is the physical exam	3.06	1.21	29.95	Disease-centered
6. When doctors ask a lot of questions about a patient’s background, they are prying too much into personal matters	4.59	0.95	87.56	Patient-centered
7. If doctors are truly good at diagnosis and treatment, how they relate to patients is not that important	4.92	1.06	88.48	Patient-centered
11. If a doctor mainly relies on being open and warm, the doctor will not have a lot of success	4.26	1.22	74.19	Patient-centered
13. A treatment plan cannot succeed if it is in conflict with a patient’s lifestyle or values	4.54	1.08	84.79	Patient-centered
14. Most patients want to get in and get out of the dentist’s office as quickly as possible	4.69	0.94	88.94	Patient-centered
16. It is not that important to know a patient’s culture and background to treat the person’s illness	4.81	0.96	88.94	Patient-centered
17. Humor is a significant ingredient in the doctor’s treatment of the patient	3.57	1.14	82.49	Patient-centered

M^a^ Mean, SD^b^ Standard deviation

**Table 4:** *t*-test and ANOVA test statistics for variables related to attitudes toward patient-centered care (N = 217)

Characteristics	N	PPOS^a^ (Overall)			PPOS (Sharing)			PPOS (Caring)		
		M^b^	SD^c^	*p*-value	M	SD	*p*-value	M	SD	*p*-value
*Gender*										
Male	137	3.82	0.42	0.28	3.38	0.48	0.27	4.27	0.58	0.48
Female	80	3.89	0.43		3.45	0.48		4.33	0.53	
*Age groups*										
20–29	26	3.71	0.33	0.11	3.32	0.36	0.58	4.11	0.52	0.09
30–39	121	3.83	0.41		3.39	0.48		4.26	0.53	
40–49	49	3.94	0.50		3.47	0.50		4.41	0.66	
50 and older	21	3.93	0.36		3.43	0.55		4.42	0.49	
*Academic track*										
Dental college	146	3.83	0.44	0.37	3.39	0.47	0.61	4.27	0.59	0.36
Professional graduate-entry school	71	3.88	0.38		3.43	0.50		4.34	0.49	
*Year of practice (y)*										
< 5	56	3.80	0.37	0.82	3.38	0.43	0.95	4.22	0.48	0.57
5–10	74	3.86	0.42		3.43	0.45		4.28	0.57	
10–15	35	3.82	0.44		3.38	0.52		4.26	0.55	
15–20	25	3.89	0.53		3.36	0.52		4.41	0.70	
20<	27	3.91	0.43		3.43	0.55		4.39	0.55	
*Affiliation*										
Primary care clinic	179	3.85	0.43	0.79	3.39	0.47	0.29	4.32	0.57	0.19
Secondary/Tertiary care hospital	38	3.83	0.40		3.47	0.49		4.19	0.53	
*Practice ownership*										
Owner/Partner	116	3.85	0.40	0.93	3.43	0.46	0.38	4.27	0.52	0.53
Associate	101	3.84	0.45		3.37	0.50		4.32	0.61	

^a^Patient–Practitioner Orientation Scale, ^b^Mean, ^c^Standard Deviation

### Predictors of patient-centered attitudes

Influencing factors on patient-centered attitudes from sociodemographic factors, academic backgrounds, work characteristics, and empathy were assessed as shown in Table 5. Work year (*β* = 0.01, *p* = 0.04) and empathic concern (*β* = 0.13, *p* = 0.04) were significant individual predictors of the overall PPOS score. The respondents’ academic track was a substantial factor in step 1 (*β* = 0.14, *p* = 0.05). However, it was no more significant after inputting empathy into the model. Similar to the overall PPOS score, work year (*β* = 0.02, *p* = 0.03), academic track (*β* = 0.18, *p* = 0.04), and empathic concern (*β* = 0.20, *p* = 0.01) were significant variables that influenced the PPOS caring subscale. Among the influencing factors, the effect of empathic concern was the strongest. Finally, for the PPOS sharing subscale, none of the demographic or empathy factors were significant.

**Table 5:** Multivariate hierarchical linear regression analysis of factors on patient-centered attitudes from among demographic and academic factors, practice ownership, and empathy

	PPOS^a^ (Total)			PPOS (Sharing)			PPOS (Caring)		
	B	ß	P	B	ß	P	B	ß	P
*Step1*									
Gender (Male:1, Female:2)	.052	.059	.400	.063	.063	.371	.041	.035	.613
Work year (y)	.011	.192	.035	.005	.070	.448	.018	.232	.011
Academic track (DC^c^:1, Professional:2)	.140	.155	.050	.073	.072	.365	.207	.174	.028
Practice ownership (Owner:1, associate:0)	− .068	− .081	.320	− .076	− .080	.330	− .061	− .054	.502
Adjusted R-squared	.013	.006	.022						
*Step2*									
Gender (Male:1, Female:2)	.043	.049	.481	.058	.059	.410	.027	.024	.727
Work year (y)	.010	.165	.067	.004	.065	.483	.015	.195	.027
Academic track (DC:1, Professional:2)	.126	.140	.074	.073	.072	.373	.179	.151	.050
Practice ownership	− .086	− .102	.205	− .082	− .086	.302	− .091	− .081	.300
IRI^b^ (Fantasy)	− .010	− .016	.839	− .009	− .012	.877	− .011	− .013	.859
IRI (Empathic Concern)	.127	.183	.035	.055	.070	.434	.200	.218	.011
IRI (Perspective Taking)	.046	.062	.459	− .019	− .023	.791	.112	.114	.165
IRI (Personal Distress)	− .009	− .013	.850	.031	.038	.587	− .050	− .052	.434
Adjusted R-squared	.042	.021	.091						

^a^PPOS Patient–Practitioner Orientation Scale, ^b^IRI interpersonal reactivity index, ^c^DC Dentistry College

## Discussion

Given the importance of PCC and effective communication in providing oral health care, our study examines dentists’ attitudes toward PCC and the factors that influence this. Significant differences emerged in dentists’ patient-centered attitudes regarding caring and sharing. Most dentists showed a patient-centered attitude toward the caring subscale. However, some showed a provider-centered attitude toward the sharing subscale. Work year, academic track, and empathy were associated significantly with a more caring aspect of patient-centered attitude, while the gender effect remained insignificant. The “empathic concern” aspect of empathy had the highest and a significant impact on the patient-centered attitude.

Several interesting findings from the results are worth noting. First, the measurement of patient-centered attitude among dentists revealed a more patient-centered philosophy in the caring aspect but more provider-centered in sharing. Of the care subscales, more than 70% of respondents disagreed with all but one item: “The most important part of the standard dental visit is the physical exam” (30%). Considering the nature of dental consultation, which involves the actual treatment process during the encounter, physical examination can be more important than general medicine. Therefore, it is reasonable to conclude that the dentist’s patient-centered attitude in caring is high.

However, regarding the sharing subscale, the proportion of the respondents who disagreed with the items varied from 16.13 to 79.3%. Most dentists favored a more provider-dominant style in seven of nine sharing items. Although there is no study comparing the scores of PPOS among dentists, more patient-centeredness in caring than sharing has been found in several previous studies [12, 13]. A qualitative study on dentists’ perception of communication revealed dentists attempted to adjust their communication to patients’ needs, treat each patient as’a whole’ rather than merely the disease itself, and build rapport with patients [13]. Regarding sharing, one study found that most dentists were aware of the benefits of involving patients in decision-making [38]. However, many dentists perceive SDM may cause patients to question their clinical expertise or unrealistic SDM because it takes too much time [12].

SDM is predicated upon patients’ good understanding of the options/alternatives for treatment and their relative advantages and disadvantages based on the evidence. However, given oral health literacy in Korea was reported to be lower than what would be considered optimal [39], patients might have difficulty participating in the decision-making process. Other studies outside Korea have also reported that dentists perceived patients’ misconceptions about treatment and treatment outcomes as problematic and an obstacle in decision-making [40]. Provider communication depends on patients’ capability to understand the dentists’ explanations and the ability to share their questions and opinions about the treatment [41]. In turn, dentists may show a provider-centered attitude toward sharing.

Second, patient-centered attitudes are positively associated with more extended dental practice experience. A more patient-centered perspective in the caring subscale was associated with longer years of work experience. This finding is fascinating, as numerous studies have shown a typical pattern of ‘ethical erosion,’ indicating the erosion of ethics-related sensibilities, empathy and patient-centeredness during clinical training increases after entering clinical rotations, residencies, or the workforce [20, 21, 31, 42, 43]. Studies of dental students show the same trend of late medical student attitudes being more doctor-centered or paternalistic than early medical students [28]. The findings confirm that the expertise of health professionals extends not only from traditional skills gained from education but also from informal clinical experience working in a particular field. Rather such competence of PCC at the dental encounter with patients cannot be simply reached through qualifications or formal education alone [44] but through practical experiences. The comprehensive clinical experience in post-graduate education contributes to practitioners’ sensitivity to patients’ needs and what they express as vital to themselves [44]. In other words, the difference in the dentists’ attitudes is likely influenced by experience and professional socialization.

Third, empathy was a key factor in dentists’ patient-centered attitudes, consistent with previous research on medical and dental students [28, 29, 45]. This study used the IRI to measure empathy in dentists and comprehensively determined that it examines four distinct aspects of empathy, including cognitive and emotional aspects. [37, 46, 47]. The affective aspect of empathy, ‘empathic concern,’ was the only significant predictor among empathy aspects. According to Hojat and his colleagues, empathic concern is the affective component of empathy, assessing ‘other-oriented’ sympathy and concern for unhappy others, and is more relevant to the context of patient care than other aspects [25, 37]. The ability to share a patient’s feelings and concerns helps dentists be attentive to the caring aspects of PCC.

### Implications of this study

A set of implications for developing interventions such as patient-centered education programs and policies to enhance PCC in dentistry can be drawn from the findings of this study. First, continuous educational interventions targeted toward increasing empathy can be effective in enhancing the patient-centered attitude of dentists [22, 48]. The finding of this study reveals dentists’ patient-centered attitude cannot be simply reached through formal education alone [44], but also from post-graduate clinical experience. Second, efforts to investigate the hurdle for implementing SDM in dental practices are urgently needed, as it can lead to a dentists’ skeptical attitude toward SDM [49–51]. For instance, concerns about delivering SDM within the health system have been revealed due to high constraints of spending enough time with patients [12]. Korean dentists are currently paid on a fee-for-services bases. Fee-for-services payment method has been widely criticized because it generally forces healthcare providers to spend less time with patients than is desirable because the fees paid per visit are too low to allow longer visits. Reform of provider reimbursement methods to create patient-centered healthcare system is highly demanded [52]. In addition, as aforementioned, patients’ poor oral health literacy can also challenge the dentist to SDM [53, 54]. Oral health education to the public as well as development and provision of patient decision aids can support SDM implementation in dental settings.

### Limitations and future directions

Several limitations should be noted in interpreting our study findings. First, we recognize that our sample was small and limited to those in only metropolitan area. The gender ratio of the respondents in this study was not very different from the actual dentists’ gender ratio, but the average age was relatively low [55]. Therefore, generalization of the present results should be taken with caution. A possible suggestion for future studies is to conduct a national-level study with more representative samples to minimize selection biases. Second, this was a cross-sectional study with no longitudinal component, making it necessary to treat comparisons between academic periods with caution, as the earlier studies in the same methodology have by nature. Third, this study did not identify predictors of patient-centered attitudes in sharing subscale. One of the potential predictors is dentists’ stress and burnout. Studies show that being a medical/dental student, resident, or physician/dentist is stressful [56, 57], and physicians experience greater burnout than the general population [58]. Cognitive and affective empathy is blunted by stressors [56, 59], which can negatively impact patient-centered attitudes. Therefore, further studies which examine potential factors, and its association with patient-centered attitudes especially sharing aspects, are advised. Further qualitative research is also recommended. To our knowledge, qualitative studies on dentists’ attitudes toward PCC are rare [13], so further qualitative studies will provide an in-depth exploration of the dentists’ attitude toward PCC in South Korea. Lastly, even though the measurement used in this study (PPOS) has been proven to have adequate reliability and validity, interpreting the results in relation to various medical situations should be taken carefully. There is a need for developing a patient-centered attitude measurement specialized for the dental setting.

## Conclusions

This study reveals patient-centered attitudes among dentists in Korea; high patient-centered attitudes in caring and relatively lower patient-centered attitudes in sharing. Efforts are needed to change attitudes toward sharing with patients and accommodate patients’ participation in decision-making. Change of patient-centered attitudes among dentists could be available through post-graduate and continuing education for dentists at the individual level and changes in the health system, encouraging shared decision-making at the system level. Moreover, personal characteristics like work experience are associated with a patient-centered attitude. This study’s results emphasize how much practice experience and professional socialization are associated with dentists’ attitude toward PCC. Therefore, continuous educational interventions targeted toward increasing patient-centered attitude are strongly advised. In addition, empathy, especially empathic concern, is a critical factor that relates to a more patient-centered attitude at dental encounters. Findings from this study support the idea that education programs should focus on enhancing empathy and conducting follow-up educational sessions to prevent students and post-graduates from becoming less patient-centered by increasing their academic or work periods.

## Supplementary Information

**Additional file**. Questionnaires.

## Data Availability

The datasets used and/or analyzed during the current study are not publicly available due to limitations of ethical approval involving the student data and anonymity but are available from the corresponding author on reasonable request.

## Abbreviations

PPOS: Patient–Practitioner Orientation Scale
PCC: Patient-centered care
SDM: Shared decision making
IRI: Interpersonal reactivity index
