# Supplementary material for: Dentists’ attitudes toward patient-centered care and its predictors: a cross-sectional study in South Korea
Source: BMC Oral Health. 2023 Feb 6;23:75. doi: 10.1186/s12903-023-02791-9 (PMC9903426; doi:10.1186/s12903-023-02791-9)
Supplement: Supplementary file 1 — Additional file. Questionnaires. [file 12903_2023_2791_MOESM1_ESM.docx]

Questionnaires

***Patient–Practitioner Orientation Scale (PPOS)***

The statements below refer to beliefs that people might have concerning dentists, patients and dental care. Read each item and then indicate how much you agree or disagree.

|  |  | Strongly agree | moderately agree | slightly agree | slightly disagree | moderately disagree | Strongly disagree |
| --- | --- | --- | --- | --- | --- | --- | --- |
| 1 | The dentist is the one who should decide what gets talked about during a visit | ① | ② | ③ | ④ | ⑤ | ⑥ |
| 2 | Although health care is less personal these days, this is a small price to pay for medical advances | ① | ② | ③ | ④ | ⑤ | ⑥ |
| 3 | The most essential part of the standard dental visit is the physical exam | ① | ② | ③ | ④ | ⑤ | ⑥ |
| 4 | It is often best for patients if they do not have a full explanation of their medical condition | ① | ② | ③ | ④ | ⑤ | ⑥ |
| 5 | Patients should rely on their dentists' knowledge and not try to find out about their conditions on their own. | ① | ② | ③ | ④ | ⑤ | ⑥ |
| 6 | When dentists ask a lot of questions about a patient's background, they are prying too much into personal matters | ① | ② | ③ | ④ | ⑤ | ⑥ |
| 7 | If dentists are truly good at diagnosis and treatment, the way they relate to patients is not that important. | ① | ② | ③ | ④ | ⑤ | ⑥ |
| 8 | Many patients continue asking questions even though they are not learning anything new. | ① | ② | ③ | ④ | ⑤ | ⑥ |
| 9 | Patients should be treated as if they were partners with the dentist, equal in power and status. | ① | ② | ③ | ④ | ⑤ | ⑥ |
| 10 | Patients generally want reassurance rather than information about their health. | ① | ② | ③ | ④ | ⑤ | ⑥ |
| 11 | If a dentist's primary tools are being open and warm, the dentist will not have a lot of success. | ① | ② | ③ | ④ | ⑤ | ⑥ |
| 12 | When patients disagree with their dentist, this is a sign that the dentist does not have the patient's respect and trust. | ① | ② | ③ | ④ | ⑤ | ⑥ |
| 13 | A treatment plan cannot succeed if it is in conflict with a patient's lifestyle or values. | ① | ② | ③ | ④ | ⑤ | ⑥ |
| 14 | Most patients want to get in and get out of the dentist's office as quickly as possible | ① | ② | ③ | ④ | ⑤ | ⑥ |
| 15 | The patient must always be aware that the dentist is in charge. | ① | ② | ③ | ④ | ⑤ | ⑥ |
| 16 | It is not that important to know a patient's culture and background in order to treat the person's illness. | ① | ② | ③ | ④ | ⑤ | ⑥ |
| 17 | Humor is a major ingredient in the dentist's treatment of the patient. | ① | ② | ③ | ④ | ⑤ | ⑥ |
| 18 | When patients look up medical information on their own, this usually confuses more than it helps | ① | ② | ③ | ④ | ⑤ | ⑥ |

Source: Krupat E, Yeager CM, Putnam S. Patient role orientations, doctor-patient fit, and visit satisfaction. Psychol Health. 2000;15:707-19.

***Interpersonal Reactivity Index (IRI)***

For each item, indicate how well it describes you by choosing the appropriate number on the scale.

|  |  | Does not describe me well |  |  |  | Describes me very well |
| --- | --- | --- | --- | --- | --- | --- |
| 1 | I daydream and fantasize, with some regularity, about things that might happen to me. | ⓞ | ① | ② | ③ | ④ |
| 2 | I often have tender, concerned feelings for people less fortunate than me. | ⓞ | ① | ② | ③ | ④ |
| 3 | I sometimes find it difficult to see things from the "other guy's" point of view. | ⓞ | ① | ② | ③ | ④ |
| 4 | Sometimes I don't feel very sorry for other people when they are having problems. | ⓞ | ① | ② | ③ | ④ |
| 5 | I really get involved with the feelings of the characters in a novel. | ⓞ | ① | ② | ③ | ④ |
| 6 | In emergency situations, I feel apprehensive and ill-at-ease. | ⓞ | ① | ② | ③ | ④ |
| 7 | I am usually objective when I watch a movie or play, and I don't often get completely caught up in it. | ⓞ | ① | ② | ③ | ④ |
| 8 | I try to look at everybody's side of a disagreement before I make a decision. | ⓞ | ① | ② | ③ | ④ |
| 9 | When I see someone being taken advantage of, I feel kind of protective towards them. | ⓞ | ① | ② | ③ | ④ |
| 10 | I sometimes feel helpless when I am in the middle of a very emotional situation. | ⓞ | ① | ② | ③ | ④ |
| 11 | I sometimes try to understand my friends better by imagining how things look from their perspective. | ⓞ | ① | ② | ③ | ④ |
| 12 | Becoming extremely involved in a good book or movie is somewhat rare for me. | ⓞ | ① | ② | ③ | ④ |
| 13 | When I see someone get hurt, I tend to remain calm. | ⓞ | ① | ② | ③ | ④ |
| 14 | Other people's misfortunes do not usually disturb me a great deal. | ⓞ | ① | ② | ③ | ④ |
| 15 | If I'm sure I'm right about something, I don't waste much time listening to other people's arguments. | ⓞ | ① | ② | ③ | ④ |
| 16 | After seeing a play or movie, I have felt as though I were one of the characters. | ⓞ | ① | ② | ③ | ④ |
| 17 | Being in a tense emotional situation scares me. | ⓞ | ① | ② | ③ | ④ |
| 18 | When I see someone being treated unfairly, I sometimes don't feel very much pity for them. | ⓞ | ① | ② | ③ | ④ |
| 19 | I am usually pretty effective in dealing with emergencies. | ⓞ | ① | ② | ③ | ④ |
| 20 | I am often quite touched by things that I see happen. | ⓞ | ① | ② | ③ | ④ |
| 21 | I believe that there are two sides to every question and try to look at them both. | ⓞ | ① | ② | ③ | ④ |
| 22 | I would describe myself as a pretty soft-hearted person. | ⓞ | ① | ② | ③ | ④ |
| 23 | When I watch a good movie, I can very easily put myself in the place of a leading character. | ⓞ | ① | ② | ③ | ④ |
| 24 | I tend to lose control during emergencies. | ⓞ | ① | ② | ③ | ④ |
| 25 | When I'm upset at someone, I usually try to "put myself in his shoes" for a while. | ⓞ | ① | ② | ③ | ④ |
| 26 | When I am reading an interesting story or novel, I imagine how I would feel if the events in the story were happening to me. | ⓞ | ① | ② | ③ | ④ |
| 27 | When I see someone who badly needs help in an emergency, I go to pieces. | ⓞ | ① | ② | ③ | ④ |
| 28 | Before criticizing somebody, I try to imagine how I would feel if I were in their place. | ⓞ | ① | ② | ③ | ④ |

Source: Kang I, Kee S-W, Kim S-E, Jeong B-S, Hwang J-H, Song J-E, Kim J-W. Reliability and validity of the Korean-version of Interpersonal Reactivity Index. J Korean Neuropsychiatr Assoc. 2009;48:352-8.
